# Effect of multimodal chemotherapy on survival of gastric cancer with liver metastasis – a population based analysis

**DOI:** 10.3389/fonc.2023.1064790

**Published:** 2023-03-16

**Authors:** Xinghui Li, Zhiqiang Chen, Yue Zhang, Hong Zhang, Haiyan Niu, Cheng Zheng, Xiaoying Jing, Hui Qiao, Guanhua Wang, Wenjun Yang

**Affiliations:** ^1^ Cancer Institute of the General Hospital, School of Public Health and Management, Ningxia Medical University, Yinchuan, Ningxia, China; ^2^ Department of Radiology, The First Affiliated Hospital, Hainan Medical University, Haikou, China; ^3^ Department of Pathology, The First Affiliated Hospital, Hainan Medical University, Haikou, China

**Keywords:** gastric cancer, liver metastases, preoperative chemotherapy, postoperative chemotherapy, palliative chemotherapy, overall survival, prognosis

## Abstract

**Objectives:**

Limited efforts have been made to evaluate the effect of multimodal chemotherapy on the survival of gastric cancer patients with liver metastases (LMGC). This study aimed to identify prognostic factors in LMGC patients and the superiority of multimodal chemotherapy with respect to overall survival (OS) in these patients.

**Methods:**

We conducted a retrospective cohort study of 1298 patients with M1 stage disease between January 2012 and December 2020. The effects of clinicopathological variables and preoperative chemotherapy (PECT), postoperative chemotherapy (POCT), and palliative chemotherapy on survival in patients with liver metastases (LM group) and non-liver metastases (non-LM group) were compared.

**Results:**

Of the 1298 patients analysed, 546 (42.06%) were in the LM group and 752 (57.94%) were in the non-LM group. The median (interquartile range) age was 60 (51–66) years. The 1-year, 3-year and 5-year overall survival (OS) rates in the LM group were 29.3%, 13.9%, and 9.2%, respectively, and those in the non-LM group were. 38.2%, 17.4%, and 10.0%, respectively (P < 0.05, > 0.05, and > 0.05, respectively.) The Cox proportional hazards model revealed that palliative chemotherapy was a significant independent prognostic factor in both the LM and non-LM groups. Age ≥55 years, N stage, and Lauren classification were also independent predictors of OS in the LM group (P < 0.05). Palliative chemotherapy and POCT were associated with improved OS compared with PECT in the LM group (26.3% vs. 36.4% vs. 25.0%, P < 0.001).

**Conclusion:**

LMGC patients had a worse prognosis than non- LMGC. Number of metastatic sites more than 1, liver and other metastatic sites, no CT treatment and HER2-negative had a poor prognosis. LMGC patient may benefit more from palliative chemotherapy and POCT than from PECT. Further well-designed, prospective studies are needed to validate these findings.

## 1 Introduction

Gastric cancer (GC) is the fourth most common cause of cancer-related death worldwide, accounting for nearly 800,000 deaths in 2020; approximately 50% of these deaths (an estimated 373,789) occurred in China (1). The poor prognosis of GC is related to local recurrence, gross peritoneal dissemination, invasion of other organs, and extensive distant metastasis (2). With current treatment strategies, patients with localised disease usually have a good prognosis, but metastatic cancers are generally incurable because of their spread to distant locations, leaving patients with no chance of radical resection and only conservative medical treatment to control the progression of the disease (3). The median survival of Chinese patients with metastatic GC varies from 3.9 months to 18.4 months based on the metastatic location (4–8). Therefore, identifying the optimal therapies for patients with metastatic GC is still needed.

The liver is a frequent site of distant metastasis in GC, with an incidence of 5–34% (9, 10). Despite significant efforts to improve survival of patients with liver metastasis (LM), the prognosis remains poor. Therapeutic surgery provides a potential cure for liver metastasis from gastric cancer (LMGC); however, surgery is currently not a standard treatment option for patients with advanced GC other than palliative surgery for bleeding, obstruction, or perforation caused by the tumour. Therefore, palliative chemotherapy is still regarded as the standard treatment modality for patients with metastatic GC (11–13). The SOPP trial showed that S-1 plus oxaliplatin (SOX) regimen can be recommended as a first-line treatment for metastatic or recurrent GC, and the median OS can be extended to12.9 months (14). According to recent studies (15–17), the median survival of patients with LMGC who undergo systemic chemotherapy is between 7 and 14 months. However, there are currently no internationally approved standard adjuvant or palliative chemotherapy regimens for patients with advanced GC. The MAGIC trial explored the preoperative administration of epirubicin, cisplatin, and infused fluorouracil (ECF) in patients with resectable GC in the United Kingdom (18) and showed that patients receiving preoperative ECF had reduced tumour size and stage and improved overall survival (OS) compared to patients treated with surgery alone (5-year OS: 36.3% vs. 23.0%, P = 0.009). Therefore, perioperative chemotherapy (PECT) is the standard treatment strategy for patients with advanced GC in Europe and the United States. The CLASSIC trial conducted at 37 centres in South Korea, China, and Taiwan showed that postoperative capecitabine plus oxaliplatin (XELOX) regimen led to better disease-free survival (DFS) than gastrectomy alone (3-year DFS, 74.0% vs. 59.0%; P < 0.001) (19), which reinforces the efficacy of POCT in GC patients in East Asia (20, 21). SOX or XELOX regimen has been recommended as one of the standard perioperative chemotherapy regimens for advanced gastric cancer in China (22–24). Nevertheless, studies on PECT and PORT in Chinese GC patients with LM was limitied.

Multimodal chemotherapy is the preferred treatment for LMGC to improve survival. However, most studies have focused on the prognosis of patients with LMGC who also undergo hepatectomy, and there are no clinical trials or cohort studies on multimodal chemotherapy in LMGC. Therefore, predicting the prognosis of LMGC patients is difficult. We retrospectively analysed the clinicopathological and survival data of LMGC patients in the Ningxia region in China, which has a high incidence of GC. We aimed to assess the clinical features and prognostic factors of LMGC to aid in identifying the optimal chemotherapy treatment timing for these patients.

## 2 Materials and methods

### 2.1 Patient population

**Figure 1:**
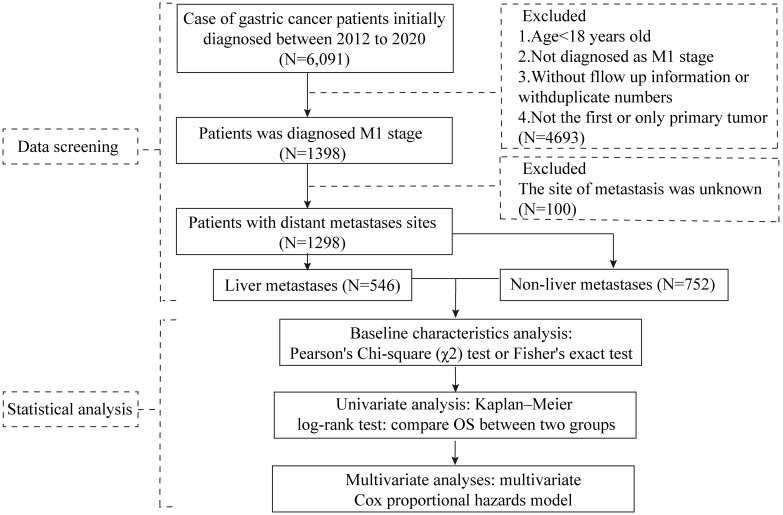
Schematic representation of the research process.

This retrospective study enrolled 1298 patients with metastatic GC from the General Hospital of Ningxia Medical University of Northwest China. The inclusion criteria were as follows (1): age ≥18 years (2) M1 stage GC diagnosed between 2012 and 2020, (3) diagnosed based on baseline radiological staging investigations, including computed tomography, performed following the initial diagnosis of GC by endoscopic biopsy and confirmed histologically, and (4) complete follow-up information. The exclusion criteria were as follows: (1) patients with duplicate numbers, (2) history of malignancy or complicating other tumours, (3) the site of metastasis was unknown. A flowchart of the patient selection process is illustrated in **Figure 1**. The study protocol was approved by the Ethical Committee of Ningxia Medical University, and informed consent was obtained from all the patients.

### 2.2 Variables

The demographic variables included age at diagnosis, sex, occupation, ethnicity, medical insurance, smoking history, alcohol consumption, family history, and blood type. Clinicopathologic variables included TN stage, primary tumour site, tumour size, differentiation, pathological type, Lauren classification, number of metastatic sites, human epidermal growth factor receptor 2 (HER2) status, serum carcinoembryonic antigen (CEA), treatment method, and survival time in months. Ethnicity was classified as Han, Hui, or others; sex was recorded as male or female; and age was grouped as <55 years and ≥55 years. The primary tumour site was classified as the upper, middle, or lower stomach. The tumour size was classified as <7 cm or ≥7 cm. The differentiation was classified into three grades: high, medium, and low. The pathological type was classified as mucinous adenocarcinoma, glandular cancer, signet-ring cell carcinoma, or other. The Lauren classification was classified as intestinal, diffuse, or mixed type. HER2 status was assessed using *in situ* hybridisation (ISH) and immunohistochemistry (IHC). HER2 was considered positive if the IHC score was 3+ and negative if the score was 0 or 1+. IHC scores of 2+ were categorised as missing if ISH was not used to confirm the result. Treatment therapies were grouped as no chemotherapy (CT), preoperative chemotherapy (PECT), postoperative chemotherapy (POCT), and palliative chemotherapy (palliative CT). The cohort was divided into two metastatic groups based on the sites of metastasis at the initial presentation: the liver metastases (LM) group (n = 546) and the no liver metastases (non-LM) group (n = 752).

### 2.3 Follow-up

All data were obtained from the patients’ medical records at the hospital. The survival information included survival status, date of death, and cause of death and was collected by telephone calls or text messages to patients or their family members. All cases were followed up in April 2021 within 2 weeks. Patients who could not be contacted after three tries or who had with incorrect phone numbers were considered lost to follow-up. Those who were lost to follow-up, alive, or died of other diseases at the end of follow-up were censored. The primary endpoint of this study was OS, which was defined as the time from the diagnosis of metastatic GC until death or the time of the last follow-up visit. Survival data were available for 558 (43.0%) patients, and follow-up information was available for 991 (76.3%) patients.

### 2.4 Statistical analysis

Descriptive statistics are presented as percentages and numerical statistics as medians. One-way analysis of variance and chi-square tests were used to compare differences in categorical variables between the groups. The Kaplan–Meier method was used to estimate OS, and the log-rank test was used to compare OS between groups. Cox regression analysis was used to calculate the hazard ratios and 95% confidence intervals (CIs) of covariates associated with OS. Factors with *P* < 0.05 in the univariate analysis were used to select covariates for the final multivariate models. Statistical significance was set at *P* < 0.05. Survival curves were plotted using GraphPad Prism (version 6.0; San Diego, California, USA). Forest plots were generated using the RMS package of R (version 4.1.0; R Foundation, Vienna, Austria). All other analyses were performed using SPSS statistical package (version 25.0; IBM Corporation, Armonk, NY, USA).

## 3 Results

### 3.1 Patient characteristics

The demographic and clinical characteristics of the patients are shown in **Table 1**.

**Table 1:** Baseline patient characteristics.

Variables	No. (%) (n=1298)	LM (%) (n=546)	Non-LM (%) (n=752)	χ²	*P*
Age (years)				25.232	<0.001
<55	438 (33.7)	142 (26.0)	296 (39.4)		
≥55	860 (66.3)	404 (74.0)	456 (60.6)		
Sex				35.773	<0.001
Male	956 (73.7)	449 (82.2)	507 (67.4)		
Female	342 (26.3)	97 (17.8)	245 (32.6)		
Ethnicity				15.967	<0.001
Han	1017 (78.4)	399 (73.1)	618 (82.2)		
Hui	265 (20.4)	140 (25.6)	125 (16.6)		
Others	16 (1.2)	7 (1.3)	9 (1.2)		
Occupation				5.853	0.119
Peasant	544 (41.9)	244 (44.7)	300 (39.9)		
Worker	322 (24.8)	140 (25.6)	182 (24.2)		
Others	168 (12.9)	61 (11.2)	107 (14.2)		
Unemployed	264 (20.3)	101 (18.5)	163 (21.7)		
BMI				15.450	<0.001
<18.5	205 (17.6)	72 (14.4)	133 (20.1)		
18.5~23	554 (47.6)	225 (44.9)	329 (49.6)		
≥23	405 (34.8)	204 (40.7)	201 (30.3)		
Cigarette smoking				4.954	0.026
No	784 (63.0)	310 (59.4)	474 (65.6)		
Yes	461 (37.0)	212 (40.6)	249 (34.4)		
Alcohol drinking				0.224	0.636
No	994 (79.9)	413 (79.3)	581 (80.4)		
Yes	250 (20.1)	108 (20.7)	142 (19.6)		
Family history				1.164	0.281
No	1168 (94.1)	485 (93.3)	683 (94.7)		
Yes	73 (5.9)	35 (6.7)	38 (5.3)		
Blood type				0.368	0.947
A	260 (29.7)	106 (29.9)	154 (29.6)		
B	269 (30.7)	106 (29.9)	163 (31.3)		
AB	87 (9.9)	34 (9.6)	53 (10.2)		
O	259 (29.6)	108 (30.5)	151 (29.0)		
T Stage				3.498	0.174
T1~T2	43 (4.0)	21 (4.6)	22 (3.5)		
T3~T4	1011 (92.9)	421 (91.3)	590 (94.1)		
Tx	34 (3.1)	19 (4.1)	15 (2.4)		
N Stage				0.882	0.643
N0	55 (5.3)	23 (5.3)	32 (5.3)		
N1~ N3	618 (59.5)	252 (57.9)	366 (60.7)		
Nx	365 (35.2)	160 (36.8)	205 (34.0)		
Differentiation				23.170	<0.001
Low	691 (70.0)	268 (62.6)	423 (75.7)		
Medium	246 (24.9)	127 (29.7)	119 (21.3)		
High	50 (5.1)	33 (7.7)	17 (3.0)		
Pathological diagnosis				29.795	<0.001
Glandular cancer	900 (88.6)	392 (91.0)	508 (86.8)		
Mucinous adenocarcinoma	27 (2.7)	5 (1.2)	22 (3.8)		
Signet-ring cell carcinoma	52 (5.1)	9 (2.1)	43 (7.4)		
Others	37 (3.6)	25 (5.8)	12 (2.1)		
Primary tumor size				1.839	0.175
<7cm	206 (45.3)	77 (49.7)	129 (43.0)		
≥7cm	249 (54.7)	78 (50.3)	171 (57.0)		
Primary tumor site				3.340	0.188
Upper	277 (26.4)	121 (27.4)	156 (25.7)		
Middle	366 (34.9)	140 (31.7)	226 (37.2)		
Lower	406 (38.7)	180 (40.8)	226 (37.2)		
Lauren’s type				12.131	0.002
Intestinal	122 (33.7)	67 (43.8)	55 (26.3)		
Diffuse	123 (34.0)	45 (29.4)	78 (37.3)		
Mixed	117 (32.3)	41 (26.8)	76 (36.4)		
Borrmann’s type				7.762	0.051
I	47 (8.2)	17 (7.9)	30 (8.5)		
II	48 (8.4)	19 (8.8)	29 (8.2)		
III	202 (35.4)	91 (42.1)	111 31.4)		
IV	273 (47.9)	89 (41.2)	184 (52.0)		
HER2				3.649	0.056
Negative	549 (88.3)	207 (85.2)	342 (90.2)		
Positive	73 (11.7)	36 (14.8)	37 (9.8)		
CEA				0.750	0.386
Negative	155 (26.7)	63 (28.8)	92 (25.5)		
Positive	425 (73.3)	156 (71.2)	269 (74.5)		
P53				2.046	0.153
Negative	108 (32.3)	44 (37.3)	64 (29.6)		
Positive	226 (67.7)	74 (62.7)	152 (70.4)		
Ki67				4.772	0.029
Low-expression	239 (40.4)	77 (34.7)	162 (43.8)		
High-expression	353 (59.6)	145 (65.3)	208 (56.2)		
EGFR				0.030	0.863
Low-expression	72 (29.1)	28 (29.8)	44 (28.8)		
High-expression	175 (70.9)	66 (70.2)	109 (71.2)		
VEGF				0.468	0.494
Low-expression	73 (31.1)	30 (33.7)	43 (29.5)		
High-expression	162 (68.9)	59 (66.3)	103 (70.5)		
Hp				0.933	0.334
Negative	213 (70.8)	105 (73.4)	108 (68.4)		
Positive	88 (29.2)	38 (26.6)	50 (31.6)		
Treatment				27.173	<0.001
No	475 (36.6)	194 (35.5)	281 (37.4)		
PECT	57 (4.4)	16 (2.9)	41 (5.5)		
POCT	391 (30.1)	140 (25.6)	251 (33.4)		
Palliative CT	375 (28.9)	196 (35.9)	179 (23.8)		
Number of metastasis sites				6.420	0.011
1	624 (48.1)	285 (52.2)	339 (45.1)		
>1	674 (51.9)	261 (47.8)	413 (54.9)		

GC, gastric cancer; LM, liver metastasis; PECT, preoperative chemotherapy; POCT, postoperative chemotherapy; CT, chemotherapy.

A total of 1298 patients with M1 stage GC were selected according to the predefined inclusion and exclusion criteria, including 956 men (73.7%) and 342 women (23.6%). The median age was 60 years (interquartile range [IQR] 51-66) and the median follow-up time was 42 months (IQR 24-61). The main histological subtype is glandular cancer (88.6%). T3 and T4 is the most common clinical stage (92.9%), and most tumours have low differentiation (70.0%). 624 (48.1%) patients have a single organ site of metastases while 674 patients (51.9%) patients have multi-organ metastases. A total of 622 patients HER2 status was assessed, including 73 (11.1%) positive patients and 549 (88.3%) negative patients. Most patients received chemotherapy (63.4%), and 34.5% of patients received surgery plus chemotherapy. Among the patients who received chemotherapy (N = 823), 57 (4.4%), 391 (30.1%), and 375 (28.9%) received PECT, POCT, and palliative CT, respectively.

To explore the relationship between metastatic patterns and survival, we divided the patients based on the presence of LM, regardless of the number of metastatic sites. Of the 1298 patients, 546 had LM and 752 had non-LM. There are 404 (74.0%) patients aged >55 years in the LM group and 456 (60.6%) patients aged >55 years in the non-LM group. There are 449 (82.2%) men in the LM group and 507 (67.4%) men in the non-LM group. There are statistically significant differences in the distributions of age and sex between the LM and non-LM groups (all P < 0.001): patients in the LM group are more likely to be older and male than those in the non-LM group. In the LM group, 16 patients (2.9%) were treated with PECT, 140 (25.6%) were treated with POCT, and 196 (35.9%) were treated with palliative CT.

### 3.2 Survival analysis

**Figure 2:**
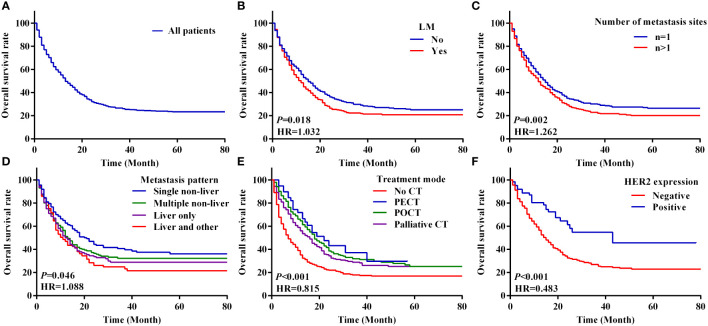
Kaplan–Meier analysis of survival curves based on clinical features with metastasis GC patients. **(A–F)** The overall survival curves of 1298 patients with M1 stage GC **(A)**, overall survival curves between the LM and non-LM groups **(B)**, number of metastasis sites of total patients **(C)**, metastasis pattern of total GC patients **(D)**, four modes of treatment **(E)**, overall survival curves between the HER2 negative and HER2 positive groups of metastasis GC patients **(F)**. LM, liver metastasis; PECT, preoperative chemotherapy; POCT, postoperative chemotherapy; CT, chemotherapy.

The median OS of all patients was 12.0 months (95% CI 10.5–13.5) and the 1-year, 3-year and 5-year OS rates were 34.4%, 15.9% and 9.6%; the survival curves are shown in **Figure 2A**. The OS rates were 25.2% and 31.4% in the LM and non-LM groups, respectively (*P* < 0.05), and the corresponding median OS were 11.0 months (95% CI 9.2–12.8) and 14.0 months (95% CI 12.0–16.0), respectively (**Figure 2B**). The 1-year, 3-year and 5-year OS rates in the LM group were 29.3%, 13.9%, and 9.2%, respectively, and the corresponding rates in the non-LM group were 38.2%, 17.4%, and 10.0%, respectively (*P* < 0.05, > 0.05, and > 0.05, respectively) (**Table 2**). Patients with single-site metastases had better survival than those with multiple-site metastases (*P* < 0.05; **Figure 2C**). In the entire cohort, 282, 264, 339, and 413 patients have liver only, liver and other, single non-liver, and multiple non-liver metastases, respectively. The OS rates were 29.4% and 23.5% for the liver only and liver and other metastases groups, respectively, both of which were significantly lower than those of the single non-liver and multiple non-liver metastases groups (39.1% and 33.2%, respectively). **Figure 2D** shows the survival curves based on the metastatic pattern.

**Table 2:** The OS of the LM and Non-LM of GC patients.

OS	LM (%)	Non-LM (%)	*P*
1-year	29.3	38.2	0.022
3-year	13.9	17.4	0.145
5-year	9.2	10.0	0.289
Total	25.2	31.4	0.015

GC, gastric cancer; LM, liver metastasis; OS, overall survival.

### 3.3 Effect of multimodal chemotherapy on survival in patients with LMGC

**Figure 3:**
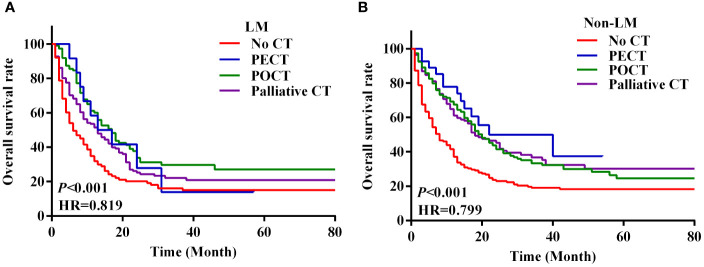
Survival curves of LM and non-LM patients with four treatment modalities. **(A, B)** Survival curves of 546 LM patients with four modes of treatment **(A)**, survival curves of 752 non-LM patients with four modes of treatment **(B)**. PECT, preoperative chemotherapy; POCT, postoperative chemotherapy; CT, chemotherapy.

We analysed the survival of patients with metastatic GC based on their characteristics and treatment modality. The survival of patients with metastatic GC was significantly different based on the chemotherapy modality. The median OS of patients who received no CT, PECT, POCT, and palliative CT were 6.0 months (95% CI 4.7–7.3), 22.0 months (95% CI 13.4–30.6), 18.0 months (95% CI 15.0–21.0), and 15.0 months (95% CI 12.4–17.6), respectively (*P* < 0.001; **Figure 2E**). In the LM group, patients who underwent POCT had the highest survival rate (36.4%). However, in the non-LM group, patients who underwent PECT had the highest survival rate (51.9%), the survival curves are shown in **Figure 3**. Notably, HER2 positivity was associated with better OS than HER2 negativity; the corresponding median OS were 43.0 months (95% CI 13.1–72.9) and 14.0 months (95% CI 12.1–16.0), respectively (**Figure 2F**). The HER2 positivity rates were 6.6% (36/546) in the LM group and 4.9% (37/752) in the non-LM group. The OS of patients with HER2 positivity were 51.7% and 68.6% in the LM and non-LM groups, respectively (P < 0.05), the survival curves are shown in **Figure S1**.

### 3.4 Prognostic factors for OS in patients with LMGC

**Figure 4:**
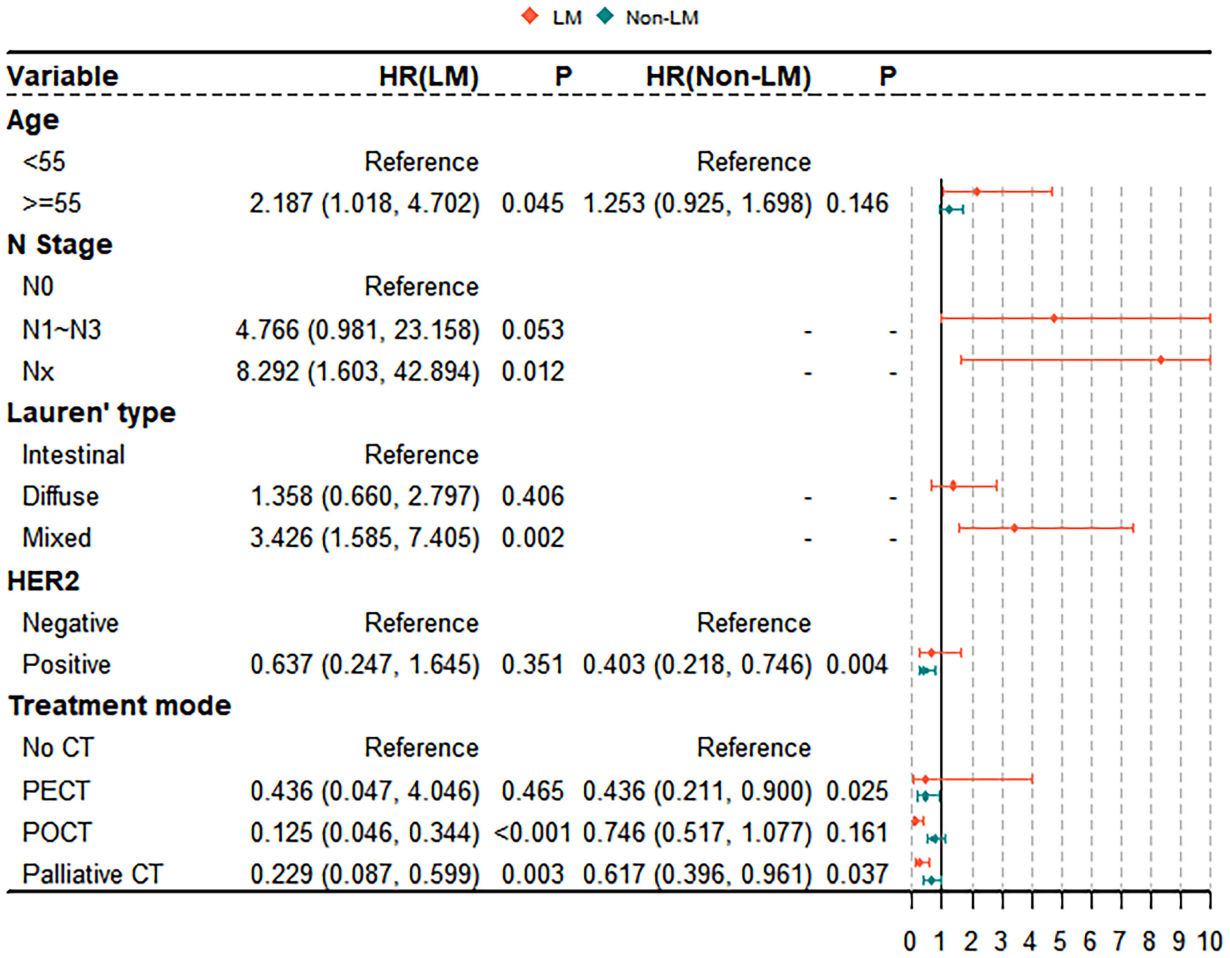
Multivariable Cox proportional hazards model for analyzing the prognostic factors for metastasis GC patients.

The results of the univariate analyses are shown in **Table S1**. Age, N stage, Lauren classification, and treatment mode are significant prognostic factors for OS in the LM group (all *P* < 0.05; **Table S1**). Age, HER2 expression, treatment mode, and number of metastatic sites are significant prognostic factors for OS in the non-LM group (all *P* < 0.05; **Table S1**). Significant variables (*P* < 0.05) associated with survival in the univariate analysis included in the multivariate Cox proportional hazards model. The results of the multivariate analyses are shown in **Figure 4**. The multivariate Cox proportional hazards model revealed that palliative CT is a significant independent prognostic factor in both the LM and non-LM groups; age ≥ 55 years, POCT, Nx stage, and mixde Lauren classification were independent predictors in the LM group, whereas PECT and HER2 positivity are independent predictors in the non-LM group. Receiver operating characteristic (ROC) was used to evaluate the accuracy of survival model, AUC=0.723, shown in **Figure S2**.

## 4 Discussion

In the present study, we retrospectively collected data of 1298 patients with M1 stage GC: 546 (42.06%) in the LM group and 752 (57.94%) in the non-LM group. All patients were from the Ningxia region in northwest China, which has a high incidence of GC. The median OS of all patients was 12 months, which was consistent with data from previous reports (median OS of 3.9–18.4 months) (4–8). Our analysis further showed that the 5-year survival rate (5ySR) of the LM group was slightly lower than that of the non-LM group (9.2% vs. 10.0%), whereas the median survival time was much lower in the LM group than in the non-LM group (11 months vs. 14 months). The 5ySR of patients with LMGC in our study is much lower than that observed in previous studies examining LMGC patients from Beijing or Japan (9.2%, 12.2%, and 31.1%, respectively) (25, 26).

Several reports, including ours, have demonstrated that old age, Lauren classification, submucosal invasion, vascular involvement, lymph node metastasis, tumour differentiation, the number of LMs, and tumour size are significant factors influencing the prognosis of patients with LMGC (27–30). In the present study, we also identified POCT and palliative chemotherapy as independent prognostic factors for patients with LMGC. Compared with those receiving PECT, GC patients with LM who received POCT and palliative chemotherapy had improved survival outcomes (25.0% vs. 36.4% vs. 26.3%, *P* < 0.001). Palliative chemotherapy is currently the most common treatment option for LMGC, and the global standard for first-line chemotherapy is 5-fluorouracil- and cisplatin-based combinations. These chemotherapies have response rates of approximately 30-40% and a median OS of 9-11 months (31), which is lower than the median OS of 15 months in LMGC patients who received palliative chemotherapy in our study. The MAGIC trial included 503 GC patients and showed that POCT after R0 resection reduced tumour size and stage, significantly improved progression-free survival and OS, and increased the 5ySR by 13%. Therefore, POCT has been clinically considered for patients with advanced GC (32). In our study, the proportions of patients receiving PECT, POCT, and palliative chemotherapy were 3.9%, 20.4%, and 34.6%, respectively. All three modes of chemotherapy improved survival in patients with LMGC compared with those who did not receive chemotherapy, and those who received POCT had the best OS (36.4%). Moreover, with the exception of palliative chemotherapy and PECT, there was no effect on the outcomes of patients without LM, which may be due to the large proportion of patients (15.2%) with more severe peritoneal metastases, which offset the effects of chemotherapy in the non-LM group. Therefore, further prospective controlled trials are needed to investigate whether palliative chemotherapy and POCT have a survival advantage in patients with metastatic GC, especially LMGC.

Some studies have demonstrated that PECT can reduce the staging of the primary tumour, increase the possibility and efficacy of radical resection, eliminate micrometastases, and prevent or reduce tumour recurrence and metastasis in GC patients (33–35). However, the survival time of patients with LMGC did not increase with PECT in our study. The reasons for the ineffectiveness of PECT in patients with LMGC are manifold. First, gastrointestinal cancers are drained by the enterohepatic circulation, reaching the liver first, which provides a rich vascular system and sufficient nutrients for the expansive growth of tumour cells (36–38). However, the majority of chemotherapeutic drugs enter the liver through the portal vein, and liver metastases affect local drug metabolism (39). Second, LMGC patients may be less responsive to chemotherapy due to the prolonged doubling time of tumour cells, and they may be unable to tolerate adequate amounts of chemotherapy drugs (40). Last, the liver microenvironment triggers inflammation, promotes angiogenesis, and enhances permeability; therefore, PECT may not be a safe and effective intervention (41). However, these speculations are based on results derived from retrospective dataset analyses involving patients with LMGC and need to be confirmed prospectively.

The results of this study support the addition of adjuvant chemotherapy to improve OS in patients with LMGC. However, this is limited to POCT, as PECT had no survival benefit. We hope that adjuvant chemotherapy can be considered as an additional therapy for patients with LMGC in the hands of rigorous surgeons and wise oncologists. Our findings, while not prospective, provide compelling motivation to explore the potential benefits of multimodal chemotherapy in patients with LMGC in randomised clinical trials.

The limitations of this study should be considered when interpreting the results. First, all participants came from one region, and the cohort may not be representative of all GC patients in China. Second, the study reviewed eight years of clinical data on patients with LMGC who received any combination of chemotherapy (administered orally, intravenously, or intraperitoneally). Patients were given SOX or XELOX perioperative chemotherapy regiments, which was divided into 2– 4 cycles, the evaluation results may be biased. Therefore, further investigation in multiple populations is required. Third, the results are dependent on the quality of data collection. In addition, there were only 57 LMGC patients in the PECT group, and the conclusion that PECT has no prognostic significance in patients with LMGC needs to be verified in larger studies.

In conclusion, this study collected 1298 patients with metastatic GC from a high GC incidence area in China, examined a variety of clinical characteristics to construct a Cox proportional hazards model, and systematically evaluated the prognostic factors that may affect patients with LMGC. Our study revealed that LMGC patients had a worse prognosis than non- LMGC. Number of metastatic sites more than 1, liver and other metastatic sites, no CT treatment and HER2-negative were all unfavorable factors to the survival of LMGC. LMGC patients who received palliative chemotherapy and POCT had significantly better survival than those who received PECT. These prognostic factors can help clinicians and patients quantify the benefits of adjuvant therapy for LMGC and make individualised treatment recommendations and decisions.

## Data availability statement

The original contributions presented in the study are included in the article/**Supplementary Material**. Further inquiries can be directed to the corresponding authors.

## Ethics statement

The studies involving human participants were reviewed and approved by Ningxia Medical University. The patients/participants provided their written informed consent to participate in this study.

## Author contributions

All the authors were involved in the design of the study or in the explanation of the data. XL, ZC and YZ together collected the data of the patients, and sorted out, analyzed and evaluated the quality of the data, which was checked by GW and WY. ZC and YZ contributed to the analysis and write-up of findings. XL wrote the first draft of the manuscript. CZ and HZ contributed to the methodology. HN, HQ and XJ contributed to proofread the article. All authors contributed to the article and approved the submitted version.
