# Supplementary material for: Effect of multimodal chemotherapy on survival of gastric cancer with liver metastasis – a population based analysis
Source: Front Oncol. 2023 Mar 16;13:1064790. doi: 10.3389/fonc.2023.1064790 (PMC10061116; doi:10.3389/fonc.2023.1064790)
Supplement: Supplementary file 1 [file DataSheet_1.docx]

Supplementary Material

# Supplementary Figures and Tables

## Supplementary Table

| Table S1 Univariate and multivariate analysis of prognostic factor associated among the mGC patients. | | | | | | | | | | | | | | |
| --- | --- | --- | --- | --- | --- | --- | --- | --- | --- | --- | --- | --- | --- | --- |
| Variables | Total (n=1298) | | LM | | | | | | Non-LM | | | | | |
|  |  |  | Univariate analysis | | | Multivariate analysis | | | Univariate analysis | | | Multivariate analysis | | |
|  | OS (%) | *P* | HR | 95% CI | *P* | HR | 95% CI | P | HR | 95% CI | *P* | HR | 95% CI | *P* |
| Age |  | <0.001 |  |  |  |  |  |  |  |  |  |  |  |  |
| <55 | 113 (36.9) |  | Reference |  |  | Reference |  |  | Reference |  |  | Reference |  |  |
| ≥55 | 167 (25.0) |  | 1.423 | 1.071~1.892 | 0.015 | 2.187 | 1.018~4.702 | 0.045 | 1.307 | 1.060~1.612 | 0.012 | 1.253 | 0.925~1.698 | 0.146 |
| Sex |  | 0.019 |  |  |  |  |  |  |  |  |  |  |  |  |
| Female | 192 (26.7) |  | Reference |  |  |  |  |  | Reference |  |  |  |  |  |
| Male | 88 (34.8) |  | 1.338 | 0.978~1.831 | 0.069 | - | - | - | 0.885 | 0.723~1.099 | 0.269 | - | - | - |
| Ethnicity |  | 0.523 |  |  |  |  |  |  |  |  |  |  |  |  |
| Han | 227 (30.0) |  | Reference |  |  |  |  |  | Reference |  |  |  |  |  |
| Hui | 48 (23.5) |  | 1.188 | 0.929~1.521 | 0.17 | - | - | - | 0.966 | 0.741~1.260 | 0.798 | - | - | - |
| Others | 5 (38.5) |  | 0.986 | 0.315~3.085 | 0.98 | - | - | - | 0.827 | 0.342~2.002 | 0.674 | - | - | - |
| Occupation |  | 0.851 |  |  |  |  |  |  |  |  |  |  |  |  |
| Peasant | 113 (28.2) |  | Reference |  |  |  |  |  | Reference |  |  |  |  |  |
| Worker | 72 (28.9) |  | 1.061 | 0.769~1.343 | 0.91 | - | - | - | 0.904 | 0.702~1.164 | 0.433 | - | - | - |
| Unemployed | 40 (32.5) |  | 0.901 | 0.606~1.341 | 0.608 | - | - | - | 0.979 | 0.717~1.336 | 0.892 | - | - | - |
| Others | 55 (27.4) |  | 1.246 | 0.927~1.675 | 0.145 | - | - | - | 0.907 | 0.692~1.188 | 0.478 | - | - | - |
| BMI |  | 0.697 |  |  |  |  |  |  |  |  |  |  |  |  |
| <18.5 | 42 (30.2) |  | Reference |  |  |  |  |  | Reference |  |  |  |  |  |
| 18.5~23 | 114 (27.9) |  | 0.967 | 0.655~1.426 | 0.865 | - | - | - | 0.925 | 0.695~1.232 | 0.595 | - | - | - |
| ≥23 | 83 (26.7) |  | 0.988 | 0.669~1.457 | 0.95 | - | - | - | 0.988 | 0.725~1.348 | 0.942 | - | - | - |
| Cigarette smoking |  | 0.722 |  |  |  |  |  |  |  |  |  |  |  |  |
| No | 174 (29.7) |  | Reference |  |  |  |  |  | Reference |  |  |  |  |  |
| Yes | 96 (27.4) |  | 1.039 | 0.823~1.311 | 0.75 | - | - | - | 1.002 | 0.810~1.238 | 0.988 | - | - | - |
| Alcohol drinking |  | 0.276 |  |  |  |  |  |  |  |  |  |  |  |  |
| No | 212 (28.3) |  | Reference |  |  |  |  |  | Reference |  |  |  |  |  |
| Yes | 58 (31.4) |  | 0.832 | 0.617~1.124 | 0.231 | - | - | - | 0.959 | 0.745~1.236 | 0.748 | - | - | - |
| Family history |  | 0.421 |  |  |  |  |  |  |  |  |  |  |  |  |
| No | 249 (28.4) |  | Reference |  |  |  |  |  | Reference |  |  |  |  |  |
| Yes | 20 (36.4) |  | 1.023 | 0.635~1.650 | 0.924 | - | - | - | 0.753 | 0.463~1.226 | 0.254 | - | - | - |
| Blood type |  | 0.655 |  |  |  |  |  |  |  |  |  |  |  |  |
| A | 49 (25.4) |  | Reference |  |  |  |  |  | Reference |  |  |  |  |  |
| B | 51 (26.3) |  | 1.099 | 0.765~1.580 | 0.61 | - | - | - | 0.845 | 0.626~1.142 | 0.273 | - | - | - |
| AB | 22 (33.3) |  | 0.732 | 0.421~1.274 | 0.27 | - | - | - | 0.867 | 0.566~1.329 | 0.513 | - | - | - |
| O | 50 (26.6) |  | 1.057 | 0.737~1.517 | 0.762 | - | - | - | 0.924 | 0.680~1.256 | 0.615 | - | - | - |
| T Stage |  | 0.061 |  |  |  |  |  |  |  |  |  |  |  |  |
| T1~T2 | 15 (41.7) |  | Reference |  |  |  |  |  | Reference |  |  |  |  |  |
| T3~T4 | 234 (30.0) |  | 1.591 | 0.869~2.914 | 0.133 | - | - | - | 1.444 | 0.769~2.711 | 0.253 | - | - | - |
| Tx | 13 (41.9) |  | 1.273 | 0.551~2.937 | 0.572 | - | - | - | 0.806 | 0.307~2.118 | 0.662 | - | - | - |
| N Stage |  | 0.073 |  |  |  |  |  |  |  |  |  |  |  |  |
| N0 | 22 (47.8) |  | Reference |  |  | Reference |  |  | Reference |  |  |  |  |  |
| N1~N3 | 25 (25.8) |  | 2.007 | 1.021~3.947 | 0.043 | 4.766 | 0.981~23.158 | 0.053 | 1.238 | 0.732~2.094 | 0.626 | - | - | - |
| Nx | 84 (28.5) |  | 2.620 | 1.322~5.191 | 0.006 | 8.292 | 1.603~42.894 | 0.012 | 1.275 | 0.743~2.189 | 0.777 | - | - | - |
| Differentiation |  | 0.384 |  |  |  |  |  |  |  |  |  |  |  |  |
| High | 160 (31.1) |  | Reference |  |  |  |  |  | Reference |  |  |  |  |  |
| Medium | 59 (28.8) |  | 1.189 | 0.728~1.941 | 0.488 | - | - | - | 2.036 | 0.820~5.057 | 0.126 | - | - | - |
| Low | 13 (35.1) |  | 1.109 | 0.693~1.774 | 0.667 | - | - | - | 2.297 | 0.946~5.580 | 0.066 | - | - | - |
| Pathological diagnosis |  | 0.3 |  |  |  |  |  |  |  |  |  |  |  |  |
| Glandular cancer | 201 (29.4) |  | Reference |  |  |  |  |  | Reference |  |  |  |  |  |
| Mucinous adenocarcinoma | 8 (32.0) |  | 0.522 | 0.129~2.101 | 0.360 | - | - | - | 0.965 | 0.574~1.625 | 0.894 | - | - | - |
| Signet-ring cell carcinoma | 13 (33.3) |  | 1.159 | 0.515~2.609 | 0.721 | - | - | - | 1.020 | 0.647~1.607 | 0.933 | - | - | - |
| Others | 13 (39.4) |  | 0.670 | 0.390~1.150 | 0.146 | - | - | - | 0.582 | 0.259~1.308 | 0.190 | - | - | - |
| Primary tumor size |  | 0.625 |  |  |  |  |  |  |  |  |  |  |  |  |
| <7cm | 49 (32.7) |  | Reference |  |  |  |  |  | Reference |  |  |  |  |  |
| ≥7cm | 136 (28.0) |  | 1.149 | 0.742~1.780 | 0.534 | - | - | - | 1.028 | 0.747~1.415 | 0.865 | - | - | - |
| Primary tumor site |  | 0.238 |  |  |  |  |  |  |  |  |  |  |  |  |
| Upper | 63 (28.9) |  | Reference |  |  |  |  |  | Reference |  |  |  |  |  |
| Middle | 101 (34.5) |  | 0.907 | 0.663~1.240 | 0.542 | - | - | - | 0.900 | 0.674~1.203 | 0.478 | - | - | - |
| Lower | 81 (27.3) |  | 0.919 | 0.683~1.238 | 0.580 | - | - | - | 1.142 | 0.857~1.522 | 0.366 | - | - | - |
| Lauren's type |  | 0.019 |  |  |  |  |  |  |  |  |  |  |  |  |
| Intestinal | 37 (39.4) |  | Reference |  |  | Reference |  |  | Reference |  |  |  |  |  |
| Diffuse | 31 (33.0) |  | 1.554 | 0.905~2.669 | 0.110 | 1.358 | 0.660~2.797 | 0.406 | 1.807 | 1.079~3.027 | 0.025 | - | - | - |
| Mixed | 26 (31.7) |  | 2.009 | 1.175~3.435 | 0.011 | 3.426 | 1.585~7.405 | 0.002 | 1.406 | 0.824~2.397 | 0.211 | - | - | - |
| Borrmann's type |  | 0.149 |  |  |  |  |  |  |  |  |  |  |  |  |
| I | 9 (23.7) |  | Reference |  |  |  |  |  | Reference |  |  |  |  |  |
| II | 16 (41.0) |  | 0.443 | 0.181~1.084 | 0.075 | - | - | - | 0.861 | 0.430~1.725 | 0.674 | - | - | - |
| III | 46 (30.3) |  | 0.725 | 0.387~1.357 | 0.315 | - | - | - | 1.002 | 0.580~1.731 | 0.994 | - | - | - |
| IV | 54 (25.2) |  | 1.010 | 0.538~1.896 | 0.974 | - | - | - | 1.158 | 0.695~1.931 | 0.573 | - | - | - |
| HER2 |  | <0.001 |  |  |  |  |  |  |  |  |  |  |  |  |
| Negative | 132 (31.3) |  | Reference |  |  | Reference |  |  | Reference |  |  | Reference |  |  |
| Positive | 39 (60.9) |  | 0.642 | 0.369~1.117 | 0.117 | 0.637 | 0.247~1.645 | 0.351 | 0.366 | 0.199~0.674 | 0.001 | 0.403 | 0.218~0.746 | 0.004 |
| CEA |  | 0.361 |  |  |  |  |  |  |  |  |  |  |  |  |
| Negative | 40 (33.6) |  | Reference |  |  |  |  |  | Reference |  |  |  |  |  |
| Positive | 95 (28.9) |  | 1.190 | 0.803~1.762 | 0.386 | - | - | - | 1.093 | 0.781~1.531 | 0.603 | - | - | - |
| P53 |  | 0.589 |  |  |  |  |  |  |  |  |  |  |  |  |
| Negative | 16 (45.7) |  | Reference |  |  |  |  |  | Reference |  |  |  |  |  |
| Positive | 33 (28.7) |  | 0.863 | 0.512~1.455 | 0.580 | - | - | - | 1.242 | 0.803~1.921 | 0.330 | - | - | - |
| Ki67 |  | 0.107 |  |  |  |  |  |  |  |  |  |  |  |  |
| Low-expression | 75 (40.1) |  | Reference |  |  |  |  |  | Reference |  |  |  |  |  |
| High-expression | 86 (32.1) |  | 1.321 | 0.895~1.948 | 0.161 | - | - | - | 1.102 | 0.817~1.487 | 0.525 | - | - | - |
| EGFR |  | 0.992 |  |  |  |  |  |  |  |  |  |  |  |  |
| Low-expression | 17 (33.3) |  | Reference |  |  |  |  |  | Reference |  |  |  |  |  |
| High-expression | 44 (32.1) |  | 1.118 | 0.591~2.117 | 0.732 | - | - | - | 0.925 | 0.561~1.527 | 0.761 | - | - | - |
| VEGF |  | 0.596 |  |  |  |  |  |  |  |  |  |  |  |  |
| Low-expression | 15 (27.8) |  | Reference |  |  |  |  |  | Reference |  |  |  |  |  |
| High-expression | 40 (31.7) |  | 0.581 | 0.456~1.552 | 0.581 | - | - | - | 0.940 | 0.578~1.527 | 0.801 | - | - | - |
| Hp |  | 0.736 |  |  |  |  |  |  |  |  |  |  |  |  |
| Negative | 46 (27.9) |  | Reference |  |  |  |  |  | Reference |  |  |  |  |  |
| Positive | 21 (30.4) |  | 0.909 | 0.547~1.512 | 0.714 | - | - | - | 0.851 | 0.543~1.334 | 0.481 | - | - | - |
| Treatment |  | <0.001 |  |  |  |  |  |  |  |  |  |  |  |  |
| No | 60 (17.5) |  | Reference |  |  | Reference |  |  | Reference |  |  | Reference |  |  |
| PECT | 17 (43.6) |  | 0.505 | 0.256~0.996 | 0.049 | 0.436 | 0.047~4.046 | 0.465 | 0.383 | 0.218~0.674 | 0.001 | 0.436 | 0.211~0.900 | 0.025 |
| POCT | 108 (36.2) |  | 0.449 | 0.333~0.605 | <0.001 | 0.125 | 0.046~0.344 | <0.001 | 0.547 | 0.432~0.692 | <0.001 | 0.746 | 0.517~1.077 | 0.160 |
| Palliative CT | 95 (32.3) |  | 0.588 | 0.453~0.764 | <0.001 | 0.229 | 0.087~0.599 | 0.003 | 0.542 | 0.418~0.703 | <0.001 | 0.617 | 0.396~0.961 | 0.037 |
| Number of metastasis sites | | 0.002 |  |  |  |  |  |  |  |  |  |  |  |  |
| 1 | 156 (32.8) |  | Reference |  |  |  |  |  | Reference |  |  |  |  |  |
| >1 | 124 (24.9) |  | 1.210 | 0.967~1.515 | 0.096 | - | - | - | 1.322 | 1.081~1.618 | 0.007 | - | - | - |

## Supplementary Figures

**

**

**Figure S1.** Survival curves of HER2 expression between LM and non-LM patients (A–B). Survival curves of HER2 expression in LM patients (A), survival curves of HER2 expression in non-LM patients (B).





**Figure S2.** ROC curve shows the sensitivity and specificity of the COX proportional hazards model in predicting the patients’ overall survival, AUC = 0.723. ROC, receiver operating characteristic; AUC, area under the curve.
